# Supplementary material for: Recent Advances of Hyaluronan for Skin Delivery: From Structure to Fabrication Strategies and Applications
Source: Polymers (Basel). 2022 Nov 10;14(22):4833. doi: 10.3390/polym14224833 (PMC9694326; doi:10.3390/polym14224833)
Supplement: Supplementary file 1 [file polymers-14-04833-s001.zip › polymers-2012105-supplementary.pdf]

Table S1. Chemical modification of HA for skin delivery.

Targeted Modification	Reaction Type	Mw (kDa)	Degree of Substitution DS (%)	Substituent	Reaction conditions	References
-COOH	Amidation	100	nr	Nanosized graphene oxide (NGO)	EDC in water (pH 5)	[118]
		20	nr	Dodecylamine	EDC/Sulfo-NHS in water/pyridine (4:1), 24 h, rt	[62]
		30	0.39–0.81	Cholesterol	EDC/ N-Hydroxy succinimide (NHS) in water/DMF	[102,119]
		300	nr	Diamino pegylated linoleic acid (PEG-LOA)	EDC/NHS mediated by DMAP in THF	[51]
		240	nr	2-dioleoyl-sn-glycero-3-phosphoethanolamine (DOPE)	EDC/NHS, water (pH 7.5), 37 °C, 24 h	[14]
		nd	nr	2,2′ (ethylenedioxy) bis (ethylamine) (diamine)	EDC in water, 24 h	[120]
		4.73	nr	Peptide ACTGSTQHQCG, poly (β-amino ester) (PAE)	NHS/EDC, double chemical modification	[104]
		10	nr	Cypate 5 and Cypate 7	DMTMM in water/DMF, 48 h at 25 °C	[48]
		10, 110	6–23	Glycerol-a-monostearate	EDC/NHS in PBS, 2 h	[17]
	Esterification	3.16	nr	Pegylated-Quercetin	80–90 °C, 8 h	[121]
		5.4	nr	Curcumin	EDC/DMAP, 50 °C for 48h	[122]
		50	0.012–0.024	Doxorubicin (DOX), Camptothecin (CPT)	DMAP and EDC in water/DMSO, 24 h, 40 °C	[123]
-OH	Esterification	15	1–5	Retinoic acid	Benzoyl chloride, DMAP, rt, 2.5 h in isopropanol alcohol/water (1:1)	[6]
		15	10	Oleic acid	Benzoyl chloride, DMAP, rt, 2 h in isopropanol/ water (1:1)	[19]
	Thiocarbamoyl bond formation	100–300	nr	Fluorescein isothiocyanate (FITC)	Dibutyltin dilaurate, $NaHCO_3$ in formamide/DMSO	[124,125]
Polymeric backbone	Reductive end modification	4.8	nr	DPPE	[$NaBH(OAc)_3$] 96 h at 60 °C, and pH 4.5	[101]

nr means that the information was non-reported by the authors

Table S2. List of selected commercial products containing-HA in European Market.

Company	Name of the product	Ingredients/technology	Cosmetic action
Clinique	100H Auto Replenishing Hydrator	Two types of HA Mw, caffeine, aloe	Improve skin hydration
L'Oréal	Revitalift Filler Hyaluronic Acid Anti-Ageing SPF50 Day Cream	Three types of HA (micro, macro, and filler)	Anti-ageing
Mario Badescu	Morning Dew Cream	Two types of HA, including hydrolysed, thyme and camellia sinensis extracts	Hydration
Drunk elephant	Lala Retro Whipped Cream	Crosslinked HA, Camellia Sinensis Seed Oil, ceramides, oil	Anti-ageing
Dr. Jart+	Cryo rubber (mask)	HA (1000 ppm)	Anti-ageing
Fresh	Lotus Face Cream	Lotus, fruits, and HA	Increases skin moisture
Iuvenio	Moon	LMW-HA grafted with retinoic acid, 9-peptide	Acti-Acne
Fresh	Rose Face Cream Reformulation	1500 and 50 kDa HA, Arabic gum, and alginate, rose extract.	Hydration effect
A-Derma	Epitheliale A.H	Cicahyalumide® (Rhealba®, a protein-free oat plantlet extract; the dipeptide L-Ala-L-Glu; and HA)	Restore fragile skin
Dr. Jart+	Ceramidin Cream	LMW-HA, beetroot.	Skin hydration
Clinique	Dramatically Different Moisturizing Cream	Glycerin, HA	Skin hydration
Dior	Hydra Life Fresh Hydration Sorbet Crème	HA, Peony extract.	Skin hydration
Dr. Jart+	Vital Hydra Solution Biome Water Cream	Hydrolyzed HA, HMW-HA Tremella Fuciformis extract.	Anti-aging.
Laniege	Water Bank Hydrating Gel	HMW-HA, Tripeptide-1, cypress extract.	Antioxidant
Hada Labo (Tokyo)	Hyaluron	Retinyl Palmitate, HMW-HA, Tocopherol, Hydrolysed collagen.	Anti-aging.
Iuvenio	Retouch serum	HA grafted with retinoic acid, Recelline.	Anti-acne.
Drunk elephant	B-Hydra™ Intensive Hydration	Crosslinked HA	Anti-ageing
Dior	Capture Totale Super Potent Serum	Aframomum angustifolium, and enriched with HA (EP1933808)	Collagen synthesis booster
The INKEY list	Retinol Serum	HA, palmitoyl Tetrapeptide-7, palmitoyl Tripeptide-1, Hydroxypinacolone Retinoate.	It slows down the breakdown of collagen
La Roche Posay	Ultra Dermallergo	Acetyl dipeptide-1-cetyl ester, HA	Decrease the dryness of skin
PIXI	Clarity Concentrate	HA, antioxidants (Liquorice, thymus)	Increased flexibility of skin.
Vichy	Lift active 10 supreme	1.5 % HA, vitamin CG	Anti-aging
	Vichy Mineral 89	Caffeine, HA, methyl gluceth-20	Reinforce the skin barrier,
Eucerin	Hyaluron-Filler	Combination of LMW and HMW-HA, Glycine, saponins and enoxolon	Anti-ageing
La Roche-Posay	Hyalu-B5 Hyaluronic Acid Serum	Vitamin B5, Madecassoside and HA	Anti-ageing

L'Oréal Paris	Revitalift Laser Pure Retinol	Retinol, HA	Anti-ageing, anti-acne.
	Revitalift 1.5% Pure Hyaluronic Acid Serum	HA, L-ascorbyl acetate	skin brightener,
Bioderma	Hydrabio (serum)	HA, apple extract, Laminaria Ochroleuca Extract,	Hydration for skin
Drunk Elephant	C-Firma Day Serum	Mixed of crosslinked and HMW-HA, Carrageenan, grape extract	Anti-ageing
Natura Bissé	Inhibit Exclusive High-Definition Serum	Mixture of HMW and LMW-HA, peptides, niacinamide, hydrolysed collagen, antioxidants	Anti-ageing
The ordinary	B5 Hydration Support Formula	HA (2%), Vitamin B5	Anti-ageing
Estée Lauder	Advanced Night Repair Intense Reset Concentrate	Mixture of HA (several Mw)	Anti-ageing
Drunk Elephant	D-Bronzi Anti-Pollution Sunshine	Crosslinked HA, colloidal nano platinum, iron oxide	Anti-ageing
Farmec	Gerovital H3 Equilibrium	Superoxide dismutase and liposomes containing -HA (10%)	Antioxidant, and anti-ageing
